# Does academic interest play a more important role in medical sciences than in other disciplines? A nationwide cross-sectional study in China

**DOI:** 10.1186/s12909-019-1737-1

**Published:** 2019-08-05

**Authors:** Hongbin Wu, Juan Zheng, Shan Li, Jianru Guo

**Affiliations:** 1 Institute of Medical Education/National Center for Health Professions Education Development, Peking University, Beijing, China; 2 Department of Educational & Counselling Psychology, McGill University, B148, 3700 McTavish St., Montreal, QC H3A 1Y2 Canada; 3 Graduate School of Education, Peking University, Beijing, China

**Keywords:** Academic interest, Academic performance, Medical sciences, College admission

## Abstract

**Background:**

Research examining the effects of academic interest on students learning achievement across various disciplines, especially a comparison of the effects of academic interest between medical sciences and other disciplines, is still scarce. This study addressed this gap by answering ‘does academic interest play a more important role in medical sciences than in other disciplines?’.

**Methods:**

A retrospective cross-sectional study, based on a large project of the National Undergraduate Student Development Survey (NUSDS) conducted by the Ministry of Education of China and Peking University in 2014, was designed to explore the role of academic interest in medical sciences and other disciplines. The participants were resampled to better represent the national distribution of undergraduate students in terms of their demographic characteristics. Specifically, survey data from 54,398 undergraduate students from 87 Chinese universities and colleges were used to address our research questions. We then used the propensity score matching (PSM) model to estimate the effect of academic interest on academic achievement and to compare the effects across different disciplines.

**Results:**

Academic interest had a significant positive impact on academic performance, with an effect size of 2.545 (*p* = 0.000). Specifically, the effect sizes for the disciplines of medical sciences, humanities, social sciences, natural sciences and engineering were 2.310 (*p* = 0.000), 2.231 (p = 0.000), 2.016 (p = 0.000), 3.840 (p = 0.000) and 2.698 (p = 0.000), respectively. The results show that no particular academic interest in medical sciences is needed to achieve academic success when compared with natural sciences and engineering programmes, but success in medical sciences requires more academic interest than success in humanities or social sciences.

**Conclusions:**

This study clarifies the effect of academic interest on undergraduates’ academic achievement while controlling for their demographic characteristics and family factors. The results provide insights into the role of academic interest in academic performance across various disciplines and can inform the college admissions practices of both institutions and high school students in China.

## Background

Medical schools select their students according to various criteria, including academic achievement, general cognitive ability, personality and interpersonal skills [1]. However, it is possible that these criteria are not sufficient to guarantee that selected students will survive their medical studies and attain professional success. For instance, a number of researchers have emphasised the role of academic interest in pursuing medicine-related studies [2]. Should academic interest be considered a crucial factor for medical schools when recruiting students? Is it true that stronger academic interest is needed to study medical sciences than for other disciplines, such as social sciences? It is likely that students choose medical schools because of the social, economic, and cultural status of medical sciences without a full awareness of their academic interests [3]. For students, the questions are as follows: Does academic interest really matter to their learning achievement? Should they be aware of their academic interests when applying to universities and disciplines, especially medical sciences? These unanswered questions urge a comprehensive study to inform college admissions practices.

Interest is generally considered to be the product of interactions between an individual and his or her environment [4]. Interest in academic domains (i.e., academic interest) is usually characterised by stable individual trends and attitudes towards academic subjects, which may yield deep engagement in learning and thus high academic performance [5]. However, research findings pertaining to the effect of academic interest on achievement are mixed due to various methodological and analytical issues [6]. Most studies have examined whether a relationship or effect exists between interest and learning achievement (e.g., grades) using either simple correlation or regression analyses. Nevertheless, many studies have quantified the relationship between interest and academic achievement without systematically controlling for alternative influence factors [5]. Very few studies have fully considered confounding factors such as school environment or family economic status. Furthermore, previous research findings are primarily limited to the selection of particular groups of participants. For example, a positive reciprocal relationship between interest and academic achievement was revealed in students from grade 5 to 7 in the domain of mathematics, whilst initial individual interest was found to have no influence on undergraduate students’ grades in an introductory psychology course [7].

The purpose of this study was to re-examine the effect of academic interest on academic achievement for a large sample of students (i.e., data collected from a nationwide survey) and to compare the effects across various disciplines while controlling for potential influence factors. It is important to consider that two major groups of potential factors could influence the relationship between academic interest and achievement: students’ demographic characteristics and family factors [5]. Taking into account the features of the Chinese educational system, we collected information pertaining to students’ characteristics, such as gender, academic track in high school, college entrance examination score, type of the university or college in which they enrolled (key versus non-key universities), method of university or college admission (first choice versus non–first choice), discipline and year of study. These factors are described below.

Notably, there are two academic tracks in high school in China: liberal arts and science. Students must choose one of these two tracks in the second of their 3 years of high school and prepare for separate national college entrance examinations. Each university or college has a unique quota for each academic track. Students can apply to any discipline they choose from medical sciences, humanities, social sciences, natural sciences, and engineering, as long as the universities or colleges provide such programmes for their corresponding academic track. Students list their preferred universities or colleges, combined with corresponding disciplines in a ranking approach (e.g., first choice – Peking University, Clinical Medicine; second choice – Tsinghua University, Educational Counselling). If a student is rejected by the first-ranked college, his or her application will be sent to the second-ranked college.

We consider the type of university and college (i.e., key versus non-key) as a crucial factor that influences students’ academic interest. This factor has never been investigated before. Key universities and colleges are those institutions that have been supported by the Chinese government to pursue world-class education and research as a national priority. They are top-ranking and highly selective. With regard to family factors, we control the influences of parental educational attainment, parental vocations, family economic status, and home location.

We then apply more sophisticated statistical procedures to eliminate the influence of the aforementioned factors. We also take a close look at the differences between medical sciences and other disciplines. Specifically, we attempt to answer the following research questions:

1. Does academic interest have an effect on academic achievement?
2. Does academic interest play a more important role in medical sciences than in other disciplines?

## Methods

### Participants and instruments

The National Undergraduate Student Development Survey (NUSDS) was conducted by the Ministry of Education of China and Peking University in 2014 to increase understanding of the growth and development of undergraduate students and the quality of higher education in China. To guarantee the reliability and validity of the survey, the NUSDS was adapted from the well-established survey instruments of the College Student Experiences Questionnaire (CSEQ) [8], Student Experience in the Research University Survey (SERU), and the National Survey of Student Engagement (NSSE) [9]. A pilot study was conducted before the large-scale quantitative research. The survey adopted a stratified sampling technique to randomly select participants based on types of institution (key versus non-key universities), discipline (medical sciences, humanities, social sciences, natural sciences and engineering) and year of study. We collected data from May 4th, 2014 to August 31st, 2014 and obtained a total of 100,941 valid questionnaires.

This investigation was a retrospective cross-sectional study with the following features: (1) we used a population-based survey to gain insights into the characteristics of undergraduate students and their development, (2) the survey was a one-time measurement and we did not alter the exposure status of the participants, and (3) the data had been collected before this study was designed. For the purpose of this study, we resampled the participants to better represent the national distribution of undergraduate students in terms of their disciplines and demographic characteristics, such as gender and home location (rural or urban areas). We used survey data from 54,398 undergraduate students from 87 universities and colleges to address our research questions; 48.4% of the respondents were male, and 31.1% were from key universities and colleges (see Table 1). As shown in Tables 1, 1920 students (3.5% of the sample) were studying medicine-related majors. The proportions of students in humanities, social sciences, natural sciences, and engineering were 19.2, 29.1, 10.1 and 38.1%, respectively. In terms of their academic track in high school, 32.3% of the students had an arts background and 67.7% had a science background. In addition, 43.5% of the students were accepted by their first-choice university or college. Overall, the population of each subgroup within the whole population of undergraduate students was adequately represented.

**Table 1:** Sample distribution and summary statistics for participants in the study (*N* = 54,398)

Variables	Sample size	Percent or Mean (SD)
Gender		
Male	26,334	48.4
Female	28,064	51.6
Disciplines		
Medical Sciences (MS)	1920	3.5
Humanities (HU)	10,426	19.2
Social Sciences (SS)	15,805	29.1
Natural Sciences (NS)	5511	10.1
Engineering (EG)	20,736	38.1
Type of Universities and Colleges		
Key Universities and Colleges	37,470	68.9
Non-key Universities and Colleges	16,928	31.1
College Entrance Examination Score	54,398	86.2 (13.4)
Academic Tracks of High School		
Arts	17,554	32.3
Sciences	36,844	67.7
Method of Admission		
First choice admission	23,661	43.5
Non-first choice admission	30,737	56.5
Grade		
Freshmen	18,043	33.2
Sophomores	14,704	27.0
Juniors	13,102	24.1
Seniors	8549	15.7
Family Factors		
ISEI of the father	54,398	30.9 (18.7)
ISEI of the mother	54,398	26.9 (15.9)
Education attainment of the father	54,398	10.5 (3.5)
Education attainment of the mother	54,398	9.2 (4.1)
Family economic status		
Low level	30,075	55.3
Middle level	18,219	33.5
High level	6104	11.2
Home Location		
Rural areas	29,963	55.1
Urban areas	24,435	44.9

Note: The umbrella category of Medical Sciences includes eight majors which are basic medicine, clinical medicine, stomatology, public health and preventive medicine, traditional Chinese medicine, integrated western and Chinese medicine, pharmacology, and science of Chinese pharmacology. *ISEI* International Socio-Economic Index of occupational status. Educational attainment is indicated by the years of education received

As mentioned above, the aim of this study was to examine the relationship between academic interest and performance while controlling as many potential confounding factors as possible. We also considered family factors. Specifically, parental educational attainment, parental vocations, family economic status, and home location were controlled in our analyses. Parental educational attainment was measured by years of education completed by the students’ fathers and mothers. As shown in Table 1, the means and standard deviations for fathers’ and mothers’ years of education completed were 10.50 (SD = 3.53) and 9.21 (SD = 4.07) years, respectively. We estimated both fathers’ and mothers’ occupational statuses using the International Socio-Economic Index of Occupational Status (ISEI) [10]. This index ranges from 16 (lowest prestige) to 88 (highest prestige). The students’ fathers’ occupational status had a mean index of 30.93 (SD = 18.71), and their mothers’ occupational status had a mean index of 26.88 (SD = 15.92). Family economic status was treated as a categorical variable with three possible values: low, middle and high. The final family factor, home location, was a categorical variable with two potential values: rural and urban.

Academic interest was assessed via the item, ‘I am interested in my academic major’. Students rated their level of agreement on a 4-point Likert scale ranging from 1 (strongly disagree) to 4 (strongly agree). We used a single item based on the following three considerations: (1) the large sample size (*N* = 54,398), (2) a single item does not overburden students, and (3) a variety of studies have demonstrated that the predictive validity of single-item measures is comparable to that of multiple-item measures [11]. We classified the students into two groups for later analyses; one group strongly agreed or agreed with the statement, and the other group disagreed or strongly disagreed with it. The reason for doing so is that we adopted a propensity score matching (PSM) approach to estimate the effect of interest (i.e., the treatment effect) on academic performance, and the treatment should be a binary variable for PSM. Thus, we transformed the variable of interest into two classes rather than four. We also asked students to report their most up-to-date cumulative grades to two decimal places. We considered the students’ grades to indicate their academic performances.

### Analyses

We used Stata MP 13.0 to explore the effect of academic interest on academic performance and to compare the effect size across disciplines (medical sciences, humanities, social sciences, natural sciences and engineering) using a PSM approach. To avoid potential estimation bias, we did not use OLS regressions because other factors that may affect academic interest, such as gender or year of study, may also influence students’ academic performance. Furthermore, OLS estimates rely on parametric assumptions, which is another limitation for data analyses. In contrast, the PSM approach accounts for variables’ effects on academic performance nonparametrically, providing more flexibility in the estimations.

We provide a brief description of the PSM approach here, and more detailed explanations were given by Rosenbaum and Rubin [12], Dehejia and Wahba [13] and Caliendo and Kopeinig [14]. The main purpose of PSM is to propose propensity scores that can adjust for the difference in covariates between treated and untreated groups. Therefore, PSM allows causal inferences to be made about treatment effects in nonexperimental observational studies [13]. Six steps are necessary for the successful implementation of PSM: choosing covariates, estimating the propensity score, choosing a matching algorithm, checking assumptions, matching the quality/effect estimations and analysing the sensitivity. For this study, we chose students’ demographic characteristics and family factors as the covariates. We estimated the propensity score of each student, based on which individuals in treatment and comparison units were matched using PSM matching algorithms (nearest neighbour, kernel matching, stratification and radius matching). We selected the algorithm that satisfied the assumptions best by testing the common support assumption, conditional independence assumption and balancing assumption. Finally, we examined the matching quality and checked the sensitivity of estimated treatment effects.

The average treatment effect on the treated (ATT) is estimated to quantify the effect of treatment on those who actually have involved in the treatment group since it is the parameter of interest in most evaluation studies [14]. Specifically, the ATT is defined as follows:

$$ ATT=E\left({y}_{1i}-{\mathrm{y}}_{0i}|\kern0.5em {w}_i\kern0.5em =\kern0.5em 1\right) $$

*y*_1*i*_ is the outcome (in this case, academic performance) of individual *i* if the individual is subjected to treatment (having academic interest), *y*_0*i*_ is the outcome of individual *i* without treatment (having no academic interest) and *w*_*i*_ is a treatment indicator, which equals 1 if individual *i* receives the treatment and 0 otherwise. Because we are interested in how the effects of academic interest on academic achievement differ across disciplines, we estimated propensity scores and ATT for each subgroup of interest.

## Results

### Effect of academic interest on academic performance

We tested the three key assumptions that underlie the use of PSM before examining the effect of interest on academic performance. The common support assumption was not violated because we collected the same types of information for all participants. The conditional independence assumption was met because we measured all covariates explicitly, and the covariates were not affected by the treatment. With respect to the last assumption, it is ideal that no significant differences exist in the covariates between the treated and untreated groups after matching. Thus, we chose the matching algorithm that satisfied this assumption best, which was the nearest-neighbour algorithm. As shown in Table 2, after matching there were no significant differences between the two groups for all covariates.

**Table 2:** Comparisons of covariates before and after matching (N = 54,398)

Variables		Mean			Reduction |bias| %	t-test	
		Treated	Control	%bias		t	p > t
Male	U	0.477	0.503	−5.3	86.1	−5.34	0.000
	M	0.477	0.473	0.7		0.97	0.333
Method of admission – First choice	U	0.596	0.497	19.9	99.5	20.04	0.000
	M	0.596	0.595	0.1		0.13	0.896
Academic track of high school-Science	U	0.652	0.728	−16.3	94.2	−16.13	0.000
	M	0.652	0.648	1.0		1.20	0.231
College entrance examination score	U	86.111	86.260	−1.1	43.0	− 1.11	0.268
	M	86.118	86.330	−1.6		−2.07	0.038
Grade-Sophomores	U	0.264	0.281	−3.9	97.1	−3.91	0.000
	M	0.264	0.264	0.1		0.15	0.881
Grade-Juniors	U	0.238	0.256	−4.1	88.6	−4.16	0.000
	M	0.238	0.240	−0.5		−0.62	0.535
Grade-Seniors	U	0.170	0.142	7.7	88.2	7.56	0.000
	M	0.170	0.173	−0.9		−1.14	0.256
Educational attainment - Fathers	U	10.594	10.319	7.8	82.3	7.83	0.000
	M	10.594	10.545	1.4		1.79	0.073
Educational attainment - Mothers	U	9.318	8.959	8.8	88.7	8.86	0.000
	M	9.317	9.277	1.0		1.30	0.193
ISEI of the father	U	31.193	30.376	4.4	66.3	4.37	0.000
	M	31.186	30.912	1.5		1.91	0.056
ISEI of the mother	U	27.106	26.312	5.0	71.5	5.01	0.000
	M	27.102	26.875	1.4		1.84	0.065
Family economic status – Middle	U	0.333	0.340	−1.4	82.2	−1.36	0.175
	M	0.333	0.335	−0.2		−0.31	0.753
Family economic status – High	U	0.114	0.107	2.0	54.2	1.98	0.047
	M	0.114	0.111	0.9		1.18	0.239
Home location – Urban area	U	0.455	0.435	4.0	74.4	4.02	0.000
	M	0.455	0.450	1.0		1.34	0.181

Note: *ISEI* International Socio-Economic Index of occupational status. *U* Unmatched, *M* Matched

Table 3 shows the treatment effect of academic interest on academic performance. In this study, the ATT measures the expected difference in academic performance between the situation in which students reported academic interest in majors and the counterfactual situation in which they did not report academic interest, while controlling for the covariates of students’ characteristics and family factors. The students’ characteristics are gender, academic track in high school, college entrance examination score, type of university and college in which they enrolled (key versus non-key), method of university or college admission (first choice versus non-first choice), discipline and year of study. The family factors are parental educational attainment, parental vocations, family economic status and home location. As shown in Table 3, academic interest has an effect on academic performance, with an effect size of 2.545.

**Table 3:** Treatment effect on academic performance

Variable	Treated	Control	Difference (ATT)	S.E.	95% CI	t	p
Academic performance	80.187	77.642	2.545	0.113	2.324–2.766	22.43	0.000

Note: the full score of academic performance is 100. *ATT* the average treatment effect on the treated, *S.E.* Standard Error, *CI* Confidence Interval for Difference (effect)

### Effect size of academic interest on performance in various disciplines

To address our second research question, we compared the effect size of academic interest on academic performance across various disciplines. We tested the aforementioned three assumptions of PSM for the subgroups of students in each of five disciplines (medical sciences, humanities, social sciences, natural sciences and engineering). The assumptions were not violated. Table 4 shows the ATT estimation results for the various disciplines. Specifically, the effect sizes of academic interest on academic performance for medical sciences, humanities, social sciences, natural sciences and engineering are 2.310, 2.231, 2.016, 3.840 and 2.698, respectively. The results reveal that students must have a relatively high academic interest to achieve academic excellence in the natural sciences. The effect of academic interest on learning achievement for medical sciences is smaller than that for natural sciences and engineering programmes; However, the effect of academic interest for medical sciences is larger than that for humanities and social sciences. Although these results are from descriptive statics, they provide foundations for asking important questions and guiding future research, such as: To what extent does the effect of academic interest account for learning achievement in medical sciences and in other disciplines? Is the effect of academic interest more important in medical sciences than that in social sciences with statistical significance?

**Table 4:** Treatment effects on academic performance in different disciplines

	MS	HU	SS	NS	EG
ATT	2.310	2.231	2.016	3.840	2.698
S.E.	0.549	0.276	0.209	0.347	0.180
95% CI	1.234–3.386	1.690–2.772	1.606–2.426	3.160–4.520	2.345–3.051
t	4.20	8.08	9.65	11.07	15.03
p	0.000	0.000	0.000	0.000	0.000
Mean Bias (%)	1.4	0.7	0.6	0.9	1.0

Note: *MS* Medicine Sciences, *HU* Humanities, *SS* Social Sciences, *NS* Natural Sciences, *EG* Engineering, *ATT* the average treatment effect on the treated, *S.E.* Standard Error, *CI* Confidence Interval for Difference (effect)

## Discussion

The findings of this study demonstrate that academic interest has a statistically significant impact on students’ academic achievement. The underlying mechanism, as suggested by previous studies, is that academic interest affects achievement through higher effort and perseverance in learning, demonstrating an add-on effect in addition to cognitive ability, which affects achievement through quicker and deep-level understanding [6, 15]. Considering the richness and completeness of the dataset used in this study, we argue that the positive effect of academic interest on learning achievement is a general principle in academic settings.

We also found that the relationship between academic interest and achievement is much stronger in natural sciences than in other disciplines. This can be explained by the fact that natural sciences are demanding subjects in which students tend to experience more negative affect, such as more anxiety and less enjoyment, than in other domains. Academic interest should therefore be particularly important to help students of natural sciences to deal with learning struggles and achieve high performance [6]. In this regard, it can be argued that the more unfavourable the characteristics of a school subject, the more important the student’s academic interest. Another explanation for this phenomenon is that academic interest affects students’ beliefs about the nature of human knowledge (e.g., whether truth is attainable or not) [16], and the influence of this belief is more evident among students who major in natural sciences than those in other domains such as social sciences [17]. Given our results, it seems that students do not perceive medical sciences to be more difficult and overwhelming than the disciplines of natural sciences and engineering. In addition, students in the majors of medical sciences may place a higher value on attributes such as responsibility, empathy and cooperation, which also have an influence on academic achievement. Therefore, the factor of academic interest does not differentiate itself from these other influencing attributes. Because this paper is one of the first to explore the effect of academic interest on achievement across disciplines, few studies are available for references or comparison. Therefore, the generalizability of the findings from this study must be confirmed by future studies. This study lays the foundations for this new stream of research.

The findings of this study have practical implications. First, academic interest affects students’ performance. It would therefore be beneficial for universities or colleges to consider academic interest as an important criterion when recruiting students. Moreover, higher education institutions could foster domain-specific interest as an effective intervention strategy to enhance students’ academic performance. Second, students should be aware of their domain-specific academic interests rather than merely relying on external factors such as occupational status or social preferences when submitting their applications to universities and colleges. For example, academic interest is of particular importance for a good performance in the ‘hard’ natural sciences. The good news for medical schools and departments is that students do not need to have a stronger academic interest in medical sciences than is required for other disciplines to gain high achievement. Nevertheless, researchers within the realm of medical sciences have previously found that the motivational profile for learning a specific curriculum (i.e., histology) differs among medical, dentistry, and pharmacy students [18]. It would be interesting to explore the effect of academic interest on achievement among students in the broad medical sciences area but with different medical backgrounds.

Although this study provides insights into the role of academic interest in achievement across various disciplines, it is not without limitations. We tried to include as many important confounding variables as possible; however, other unmeasured or unobserved variables cannot be accounted for using PSM. For example, it is possible that students’ personalities, learning styles, and motivational preferences (e.g., fulfilment, power, control, or public recognition) also play a role in determining their academic performance, whether directly or indirectly. Like Clark and Duwe [19], we were unable to measure or control for everything. Furthermore, self-report studies generally suffer from specific limitations. For example, participants may not be able to recall the details of their responses in previous events, or they may be reluctant to reveal private information. We used a single item to measure students’ interest, which could undermine the validity of the measurement, although we had a very large sample size and the practice of using a single item has been shown to be effective in various studies [11]. Moreover, it is unclear whether high interest results in high academic performance or if high academic performance strengthens an individual’s interest. Thus, one direction for our future research will be to explore the interplay of interest and academic performance using a longitudinal approach by collecting the same types of information for multiple years and examining the reciprocal relationships between variables in cross-lagged panel models.

## Conclusions

This study examined the relationship between academic interest and achievement using a comprehensive large-scale dataset, the National Undergraduate Student Development Survey, from which we obtained data for 54,398 undergraduate students from 87 Chinese universities and colleges. The results demonstrate that academic interest does yield a positive effect on achievement, and the relationship between academic interest and achievement is much greater in the natural sciences than in other disciplines. The findings suggest that medical schools should value but not overemphasise the role of academic interest in students’ professional learning. This study contributes significantly to the growing body of research pertaining to academic interests in at least three ways. First, it informs the practice of college admissions in China in terms of academic interest for both institutions and high school students. Second, we considered students’ background characteristics and family factors and controlled for as many confounding variables as possible with a PSM approach. Finally, we examined the effect of academic interest on achievement across various disciplines, which has rarely been done in prior studies.

## Data Availability

The datasets used and/or analysed during the current study are available from the corresponding author on reasonable request.

## Abbreviations

ATT: The Average Treatment effect on the Treated
CSEQ: College Student Experiences Questionnaire
EG: Engineering
HU: Humanities
ISEI: International Socio-Economic Index of Occupational Status
MS: Medicine Sciences
NS: Natural Sciences
NSSE: National Survey of Student Engagement
NUSDS: National Undergraduate Student Development Survey
PSM: Propensity Score Matching model
SERU: Student Experience in the Research University Survey
SS: Social Sciences
